# Correction: Physiotherapy and associated factors affecting mouth opening changes in noma patients during initial hospitalization at an MSF-supported hospital in Northwest Nigeria—A retrospective cohort study

**DOI:** 10.1371/journal.pgph.0002879

**Published:** 2024-01-24

**Authors:** Oluwole Victor Oluwalomola, Emily Briskin, Michael Olaleye, Joseph Samuel, Bukola Oluyide, Mark Sherlock, Adeniyi Semiyu Adetunji, Mohana Amirtharajah

The Funding statement is incorrect. The correct statement is: This operational research was funded through the authors’ routine Médecins Sans Frontières (MSF) country programme in Nigeria and specifically through the noma project in Sokoto, Nigeria. No grants were part of this study. MSF provided support in the form of salaries for all authors. The funder had no role in study design, data collection and analysis, decision to publish, or preparation of the manuscript.
