# Supplementary material for: Physiotherapy and associated factors affecting mouth opening changes in noma patients during initial hospitalization at an MSF-supported hospital in Northwest Nigeria—A retrospective cohort study
Source: PLOS Glob Public Health. 2023 Sep 1;3(9):e0001995. doi: 10.1371/journal.pgph.0001995 (PMC10473475; doi:10.1371/journal.pgph.0001995)
Supplement: Attachment — Submitted filename: Response to Reviewers.docx [file pgph.0001995.s002.docx]

Oluwole Victor Oluwalomola

Email: Oluwolevctr@gmail.com

Dear Dr Javier H Eslava-Schmalbach,

I am writing in relation to the revised manuscript “Physiotherapy and associated factors affecting mouth opening changes in noma patients during initial hospitalization at an MSF-supported hospital in Northwest Nigeria” (PGPH-D-22-01615). Thank you for the acceptance of this manuscript for publication in PLOS Global Public Health Journal.

We have addressed each of the comments and included all the changes in the manuscript.

Please find below the responses to each point raised by the editor and reviewer(s).

1. Add a statement mentioning the normal distribution of the dependent variable;

Response: Thank you for this comment. We have added into the boxplot in Figure 1 a box representation of the dependent variable (change in mouth opening) to demonstrate the distribution of the dependent variable.

2. Clarify why you sometimes use the median and other times the mean to describe the behavior of the data (Table 1 and the following paragraph);

Response: Thank you for this comment. For consistency across the tables in the result chapter we took out the median and used the mean to describe the data in the tables and the corresponding paragraph.

3. Mention if other assumptions needed to run linear regression models were tested and;

Response: Thank you for this comment. We revised the previous statement which stated that assumptions were checked for multivariate analysis, we added that all assumptions to be met for linear regression and multivariate analysis were checked and tested.

4. Introduce the meaning of the acronyms when you mention them for the first time.

Response: Thank you for this comment. The meaning of every acronym mentioned for the first time have been rightly inputted.

Sincerely,

On behalf of the author group,

Oluwole Victor Oluwalomola (corresponding author)

oluwolevctr@gmail.com
