# Supplementary material for: Physiotherapy and associated factors affecting mouth opening changes in noma patients during initial hospitalization at an MSF-supported hospital in Northwest Nigeria—A retrospective cohort study
Source: PLOS Glob Public Health. 2023 Sep 1;3(9):e0001995. doi: 10.1371/journal.pgph.0001995 (PMC10473475; doi:10.1371/journal.pgph.0001995)
Supplement: Attachment — Submitted filename: Response to Reviewers..docx [file pgph.0001995.s003.docx]

Oluwole Victor Oluwalomola

Email: Oluwolevctr@gmail.com

Dear Dr Javier H Eslava-Schmalbach,

I am writing in relation to the revised manuscript “Physiotherapy and associated factors affecting mouth opening changes in noma patients during initial hospitalization at an MSF-supported hospital in Northwest Nigeria” (PGPH-D-22-01615R1). Thank you for the acceptance of this manuscript for publication in PLOS Global Public Health Journal.

We have addressed each of the comments and included all the changes in the manuscript.

Please find below the responses to each point raised by the editor.

1. Changes to reference list

Response: Thank you for this comment. We have added a doi for reference number 1 and replaced reference number 10 with a relevant current reference.

2. Submission of article to Research Article

Response: Thank you for this comment. The revised manuscript have been appropriately submitted to research article.

3. Addition of type of study

Response: Thank you for this comment. We have added the type of study to the end of the study title has advised.

Sincerely,

On behalf of the author group,

Oluwole Victor Oluwalomola (corresponding author)

oluwolevctr@gmail.com
